# Origin, activation, and targeted therapy of glioma-associated macrophages

**DOI:** 10.3389/fimmu.2022.974996

**Published:** 2022-10-06

**Authors:** Can Xu, Menglin Xiao, Xiang Li, Lei Xin, Jia Song, Qi Zhan, Changsheng Wang, Qisong Zhang, Xiaoye Yuan, Yanli Tan, Chuan Fang

**Affiliations:** ^1^ School of Clinical Medicine, Hebei University, Department of Neurosurgery, Affiliated Hospital of Hebei University, Baoding, China; ^2^ Hebei Key Laboratory of Precise Diagnosis and Treatment of Glioma, Baoding, China; ^3^ Hebei University School of Basic Medical Sciences, Department of Pathology, Affiliated Hospital of Hebei University, Baoding, China; ^4^ Tianjin Key Laboratory of Composite and Functional Materials, School of Material Science and Engineering, Tianjin University, Tianjin, China

**Keywords:** Glioma, glioma associated macrophages (GAMs), recruitment, polarization, therapy resistance, targeted therapy

## Abstract

The glioma tumor microenvironment plays a crucial role in the development, occurrence, and treatment of gliomas. Glioma-associated macrophages (GAMs) are the most widely infiltrated immune cells in the tumor microenvironment (TME) and one of the major cell populations that exert immune functions. GAMs typically originate from two cell types-brain-resident microglia (BRM) and bone marrow-derived monocytes (BMDM), depending on a variety of cytokines for recruitment and activation. GAMs mainly contain two functionally and morphologically distinct activation types- classically activated M1 macrophages (antitumor/immunostimulatory) and alternatively activated M2 macrophages (protumor/immunosuppressive). GAMs have been shown to affect multiple biological functions of gliomas, including promoting tumor growth and invasion, angiogenesis, energy metabolism, and treatment resistance. Both M1 and M2 macrophages are highly plastic and can polarize or interconvert under various malignant conditions. As the relationship between GAMs and gliomas has become more apparent, GAMs have long been one of the promising targets for glioma therapy, and many studies have demonstrated the therapeutic potential of this target. Here, we review the origin and activation of GAMs in gliomas, how they regulate tumor development and response to therapies, and current glioma therapeutic strategies targeting GAMs.

## Introduction

Gliomas are a group of primary brain tumors of glial tissues that include astrocytomas, oligodendrogliomas, and glioblastomas (GBMs). Among them, GBM is the most commonly occurring malignant primary brain carcinoma. Even with the standard combination treatments involving surgical resection, postoperative radiotherapy, and chemotherapy, the median survival time of GBM patients is only 14.6 months. Besides, tumor recurrence and death are almost inevitable in GBM patients (1). The development of drug-resistance glioma and difficulties in designing effective treatment strategies are largely due to the high degree of heterogeneity in tumor-associated cellular and genetic signatures as well as to the preventive actions of the blood-brain barrier (BBB) (2, 3). Furthermore, the infiltration of glioma-associated macrophages (GAMs), T-regulatory cells (Tregs), and myeloid-derived suppressor cells (MDSCs) in the tumor microenvironment (TME) greatly contributes to the pathogenesis of complex malignant phenotypes and impairment of antitumor immune functions (4). In addition to the tumor cells, gliomas also contain many non-tumor infiltrates that mediate the tumor initiation, progression, and response to therapies. Most non-tumor cells in gliomas are GAMs, recruited to the glioma microenvironment under homeostatic and/or inflammatory conditions. GAMs are immunologically active and can modulate the release of various growth factors and pro-inflammatory cytokines, which generate a supportive matrix for the metastasis of tumor cells and promote the formation of an immunosuppressive TME of gliomas (5). The glioma microenvironment is characterized by high levels of immunosuppressive cytokines and significant populations of Tregs and bone marrow-derived macrophages (BMDMs) (6). In gliomas, the number of GAM infiltrates positively correlates with the glioma grading by the World Health Organization (WHO) and negatively correlates with patient survival (7, 8). Increased peritumoral BMDM infiltration has been observed in GBM patients (9). Moreover, monocytes from healthy donors have been found to acquire BMDM characteristics after treatment with a conditioned medium of a GBM cell line (10). Further explorations of GAMs and the tumor immune microenvironment are fundamental to developing novel immunotherapeutic strategies for glioma management (11).

**Figure 1:**
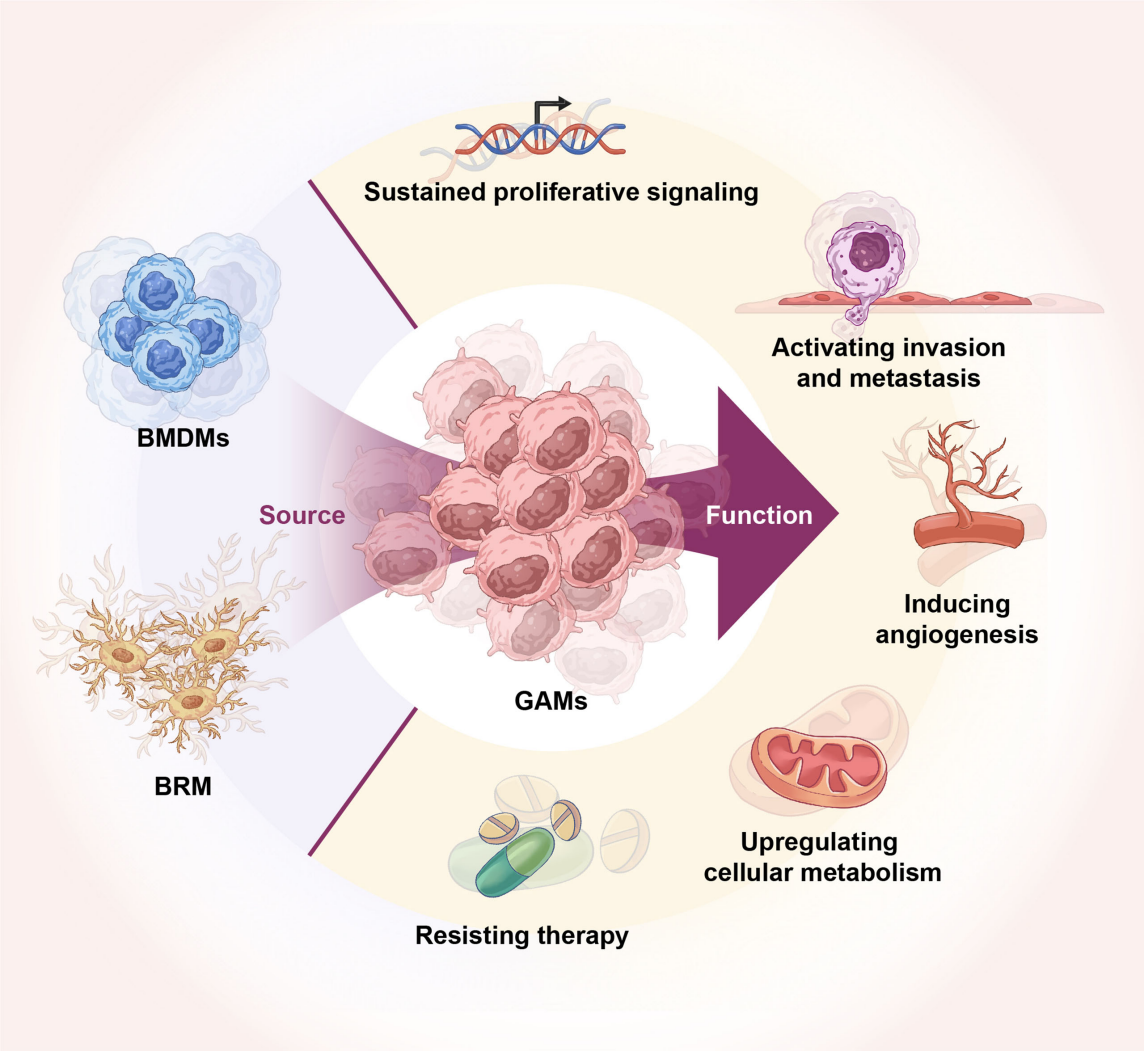
Origin of GAMs and their role in glioma. GAMs originate from BRM and BMDMs. GAMs regulate glioma tumor growth, invasion, angiogenesis, energy metabolism, and therapy resistance.

Due to the existence of BBB, the brain has always been considered a unique immune organ (12). However, the specificity of the brain immune system is viewed as more immunologically different than an immune-specific organ (13). Gliomas originate from the primary neural stem or glial cells in the central nervous system (CNS), and the glioma microenvironment is unique in the sense that it contains a mixed population of neurons, astrocytes, resident myeloid cells, and microglia. Glioma-specific GAMs include brain-resident macroglia (BRM) or BMDM (14, 15). GAMs are the most widely infiltrated immune cells in the glioma microenvironment, mediating diverse and complex functions such as tumor metastasis, angiogenesis, treatment resistance, and development of an immunosuppressive microenvironment (**Figure 1**) (16). The importance of the GAM population for glioma development is reflected in the fact that GAMs account for 50% of all cells in human GBM (17, 18). There is also a marked infiltration of myeloid cells in high-grade gliomas, accounting for more than 85% of GAMs within gliomas are BMDMs, whereas BRMs predominate in the peri-tumoral areas (18, 19).

Although the activation states of GAMs are complex, however, it is often presented into two categories the simplicity-classically activated macrophages or M1 macrophages and alternatively activated macrophages or M2 macrophages, which exhibit antitumor/immunostimulatory and protumor/immunosuppressive effects, respectively (20). Postnatal development of macrophages occurs through the macrophage colony-stimulating factor (M-CSF) or granulocyte-macrophage colony-stimulating factor (GM-CSF)-dependent differentiation of circulating monocytes, which originate from bone marrow-derived progenitor cells (21). Previous studies have shown that the M1-type macrophages, activated by the increased exposure to stimuli like GM-CSF, interferon-gamma (IFN-γ), tumor growth factor alpha (TGF-α), and lipopolysaccharide (LPS), express surface markers such as CD68, CD80, CD86, and secretory cytokines including IL1β, IL6, IL12, IL23, CXCL9 and CXCL10 (22); while M2-type polarization is closely related to the levels of M-CSF, IL4, IL10, IL13, TGF-β and glucocorticoids, and activated M2 macrophages express surface markers like CD163, CD204, and CD206 as well as secretory cytokines including IL10, TNF-α, CCL17, CCL18, CCL22, and CCL24 (22, 23).. The primary strategy of GAM-targeted therapy for glioma aims to reduce the recruitment of BMDM and reprogram GAMs from the M2 to M1 phenotype, thereby reversing the characteristics of the anti-inflammatory tumor immune microenvironment (16). In this review, we summarize the origin, recruitment, and activation mechanisms of GAMs in gliomas, regulatory factors for the M1/M2 polarization of GAMs, and the effects of GAMs on glioma development and therapeutic outcomes, as well as the research progress on GAM-targeted treatment strategies.

## Origin and activation of GAMs

### Origin of GAMs

Glioma-associated macrophages originate from two types of cells, namely BRM and BMDMs (24). Florent Ginhoux et al. have shown for the first time that the resident microglia in the brain originate from the extraembryonic yolk sac cells (25). Katrin Kierdorf et al. have further demonstrated that mouse microglia could be derived from the primitive c-KIT-positive erythrocyte precursors (26). In the adult brain, microglia perform multiple tasks such as functional support to neurons, phagocytosis of apoptotic cells, and immune surveillance (27, 28). Moreover, the microglia pool is maintained by their self-renewal mechanisms without the contribution of myeloid-derived progenitor cells. Therefore, microglia are the only resident immune cell population in the healthy brain (29, 30). Microglia can be discriminated from GAMs with the microglia-specific genes P2RY12 and TMEM119, higher proportion of microglia may be beneficial for patient survival in glioblastoma (31). Monocytes originate from progenitor cells in the bone marrow. During homeostasis and inflammation, circulating monocytes leave the bloodstream and migrate into tissues, where they differentiate into macrophages or dendritic cell populations depending on the presence of local growth factors, pro-inflammatory cytokines, and microbial products (32). In gliomas, the local inflammatory milieu compromises the integrity of the BBB, permitting the infiltration of inflammatory monocytes into the brain from the circulation, which then differentiates into bone marrow-derived GAMs (5) mediating the inhibition of tumor-specific immune defense mechanisms (33). However, some researchers have put forward a different point of view. Alexander Mildner et al. have used a set of bone marrow chimeric and adoptive transfer experiments to show that BRM can be derived by the differentiation of circulatory LY6C^HI^CCR2^(+)^ monocytes after being infiltrated into brain lesions under pathological conditions. Under the diseased conditions, microglial engraftment in the CNS is not associated with the BBB disruption but rather requires prior brain modulation (e.g., direct tissue irradiation). Experimental results have identified LY6C^HI^CCR-2^(+)^ monocytes as the immediate precursors of microglia in the adult brain and clarified the importance of local stimulatory factors for the microglial engraftment in the adult CNS (34).

### Characteristic differences between GAMs

In the past, CD45 expression was usually measured to distinguish BRM (low expression level) from BMDM-derived GAMs (high expression level). Immunofluorescence (IF) analysis of patient-derived glioma samples has shown that macrophages with high CD45 expressions are significantly more abundant than those with basal or low expressions (35). However, this notion has been challenged in recent years. New studies have indicated that although the CD45 expression level can differentiate BRM- from BMDMs-derived GAMs in mice, cell-type-specific CD45 expression profiles differ between mice and humans. Moreover, CD45 expression level could not accurately distinguish BRM- from BMDMs-derived GAMs in glioma patient samples, suggesting the need for more sensitive and specific RNA-sequencing, flow cytometry, and other comprehensive analyses to distinguish further the gene expression differences between BRM- and BMDM-derived GAMs (36).

A large-scale RNA-sequencing analysis revealed differential gene expression patterns specific to infiltrating and resident cells, suggesting that populations of GAMs from different origins may perform distinct functions (18). One study has identified the marker protein transmembrane protein 119 (TMEM119) specifically and stably expressing only in BRM-derived GAMs. Further RNA sequencing based on the TMEM119 expression profile has demonstrated unique differences in the BRM- and BMDM-derived GAM transcriptomics. The study has also reported that the gene expression pattern of BRM could be different at different developmental stages. As the microglia matures, the expression of its uniquely expressed genes (e.g., *TMEM119*, *P2RY12*, and *OLFML3*) increases but the cell proliferation ability decreases (37). Another study has utilized multiple genetic lineages for tracing different glioma models. Transcriptional network analysis has identified the modulator ITGA4 (CD49d) that mediates the differentiation of BRM- and BMDM-derived macrophages under a homeostatic condition. Besides, these macrophages express genes related to their specific biological functions (36). Sören Müller et al. conducted a single-cell RNA sequencing (scRNA-seq) analysis of clinical glioma specimens and identified a novel genetic signature. Two markers, CD49d and P2RY12, are detected to differentiate between BRM- and BMDM-derived GAMs under both malignant and non-malignant conditions. Compared with microglia, myeloid-derived monocytes can upregulate the expression of immunosuppressive cytokines, markers of M2 activation (like IL10 and TGF-βII), phagocytosis, and activated tricarboxylic acid (TCA) cycle (38). Notably, these gene expression and functional differences between BRM- and BMDM-derived GAMs are present in both human and mouse GAMs (36–38).

Additionally, the immune compartments within the glioma microenvironment are characterized by a level of heterogeneity and dynamicity of immune cells that cannot be recapitulated by the simplistic paradigm of M1 and M2 phenotypes. Ana et al. employed scRNA-seq and CITE-seq to map the GBM immune landscape in mouse tumors and in patients with newly diagnosed disease or recurrence. BRM- and BMDM-derived GAMs are self-renewing cell populations that compete for space and can be depleted by CSF1R blockade. Microglia-derived GAMs dominate in newly diagnosed tumors but are overtaken by monocyte-derived TAMs after recurrence, especially in hypoxic tumor environments (39). Furthermore, Natalia et al. performed scRNA-seq on microglia, monocytes, and macrophages in male and female mouse gliomas to identify distinct transcriptional programs in GAMs, the findings suggest that glioma-activated microglia Sex-specific genes (MHCII and *CD74*) expression in cells may be associated with morbidity and outcome in glioma patients (40). Single-cell analysis by Nourhan Abdelfattah et al. has defined the BMDM as one of nine subtypes with spatial heterogeneity, representing distinct immune states, and nominated S100A4 as a promising immunotherapy target (41).

### Recruitment and activation mechanisms of GAMs

#### Classification of monocyte subsets

Monocytes are divided into subpopulations based on their differences in the expression profiles of chemokine receptors (CCRs) and specific surface molecules (42, 43). In mice, monocytes can be categorized into two subtypes based on the expression of *LY6C* and *CX3CR1* genes, such as LY6C^HI^ and LY6C^LOW^ (also known as CX3CR1^HI^) monocytes. The LY6C^HI^ monocytes are called inflammatory monocytes, which typically express high CCR2 but low CX3CR1 levels, occupying approximately 2-5% of circulating leukocytes in healthy mice, and are rapidly recruited to sites of infection and inflammation (44). The role of CCR2 is critical in monocyte trafficking, and the deletion of this chemokine receptor significantly reduces the trafficking of LY6C^HI^ monocytes toward the inflammation sites (45, 46). While LY6C^LOW^ monocytes are less populated than LY6C^HI^ monocytes and typically express high levels of CX3CR1 and low levels of CCR2 and LY6C (42, 43). *In vivo* microscopy studies have shown that LY6C^LOW^ monocytes adhere and migrate along the luminal surface of endothelial cells in small blood vessels, a process called patrolling (47). In humans, monocytes are classified into three subtypes based on the differential expression of monocyte-specific antigens CD14 and CD16- classical (CD14^++^CD16^-^), non-classical (CD14^+^CD16^++^), and an intermediate (CD14^++^CD16^+^) subtypes (48). Classical monocytes, similar to those of mouse LY6C^HI^ monocytes, highly express CCR2 and are the most prevalent monocyte subset in human blood (49). Non-classical monocytes resemble the mouse LY6C^LOW^ monocyte population and perform patrolling functions *in vivo*. Although the monocyte subtypes identified in humans and mice are not identical, their process of differentiation and roles in immune defense mechanisms appear to be similar (50–52).

#### Chemokines and recruitment of BMDMs

Chemokines are the largest subfamily of cytokines, best known for their roles in directing the movement of immune cells throughout the body (53). According to the position of the first two cysteine ​​(C) residues in their protein sequences, chemokines are classified into four subclasses- CC, CXC, CX3C, and XC (54). Chemokine ligand 2 (CCL2) (previously known as MCP1) is the most crucial member of the CC chemokine family. CCL2 and its receptor (CCR2) are involved in regulating the monocyte/macrophage migration from the blood circulation to the brain through the vascular endothelium and are key pathogenic factors in glioma progression (55). CCL2 and CCL7 mediate the recruitment of bone-marrow-derived LY6C^HI^CCR2^+^ monocytes *via* binding to CCR2. Furthermore, CCL2 is responsible for the recruitment of CCR4-expressing Tregs to the glioma microenvironment (56). Glial cells and macrophage-derived (especially CD163^+^ macrophages) CCL2 is an independent prognostic factor in GBM patients, and the CCL2-CCR2/4 axis is a potential GBM immunotherapy target (57). Loss of CCL2 or CCL7 can significantly reduce the recruitment of myeloid-derived monocytes during inflammation by approximately 40–50% (58). One possible mechanism by which CCL2 recruits BMDM to the TME could be the binding of circulating CCL2 molecules to glycosaminoglycans in gliomas to establish a concentration gradient that guides monocytes toward the site of inflammation. Another possibility could be that CCL2 and CCL7 may act in tandem to direct monocytes to lesion sites in the bone marrow, or they may act in parallel to drive the monocyte recruitment from distinct regions within the bone marrow or other tissues to the TME (59, 60).

Moreover, it has been reported that Duffy antigen receptor chemokine (DARC), which binds CCL2 and transports it to the medial lumen through the endothelium, is essential for the recruitment of monocytes from the blood to inflamed tissues (61). CCL8 and CCL12 also bind CCR2, but deletion of the genes encoding these chemokines has no detectable effect on monocyte trafficking under homeostasis conditions (56). Additionally, studies have shown that activation of the CX3CL1-CX3CR1 signaling pathway can enhance the accumulation of GAMs and promote angiogenesis during the malignant transformation (62). Activated CXCL2-CXCR2 signaling recruits and activates BRM/macrophages through the activation of extracellular signal−regulated protein kinase 1 and 2 (ERK1/2) and AKT signaling pathways, thereby promoting the GBM progression (63).

#### Adhesion molecules associated with the BRDM transportation

Monocyte recruitment is thought to follow the general leukocyte adhesion and transportation model, including rolling, adhesion, and migration. The migration of leukocytes, including monocytes, depends on the integrins and several other adhesion molecules (64). Monocytes of LY6C^HI^ mice expressing selectin L (also known as CD62L), selectin P glycoprotein ligand 1 (PSGL1), integrin αLβ2 (also known as LFA1), integrin αMβ2 (also known as MAC1), platelet-endothelial cell adhesion molecule (PECAM1), and integrin α4β1 (also known as VLA4) contribute to leukocyte adhesion and migration. The patrolling of monocytes along the resting dermal vascular endothelium in LY6C^Low^ mice is shown to be mediated by the integrin αLβ2 (47). In contrast, early recruitment of LY6C^HI^ monocytes is not affected by αLβ2 deficiency (65). Additionally, POSTN, an extracellular matrix component produced by glioma stem cells, provides an efficient binding site for αVβ3 integrins on the cell surface of peripheral monocytes and M2-GAMs to promote the extravasation and migration in the glioma microenvironment (5). Besides, carbonic anhydrase XI (CAIX) promotes macrophage adhesion to glioma cells, cell motility, and macrophage polarization (66).

#### Recruitment and activation of GAMs mediated by other regulators

Some other proteins, amino acids, and cytokines have also been reported to be correlated with the recruitment and activation of GAMs. Studies by Quan Zhang et al. have demonstrated that overexpression of programmed cell death protein 10 (PDCD10) promotes the release of CXCL2 and activates CXCR2 and ERK1/2-mediating signaling, thereby recruiting and activating BRM/macrophages to promote the GBM progression (63). Zhuo Chen et al. have found that farnesyl diphosphate synthase (FDPS) can trigger the Wnt/β-catenin signaling pathway and promote the infiltration of GAMs by inducing the expression of CCL20 (67). The bifunctional cytokine IL33 secreted by GBM cells in humans and mouse positively correlates with the tumorigenic infiltration of GAMs. Secreted IL33 functionally regulates chemokines that co-recruit and activate circulating and resident innate immune cells. Moreover, IL34 acts through the colony-stimulating factor 1 receptor (CSF-1R) on the surface of peripheral monocytes that mediate monocyte attachment to the vascular endothelial layers (68, 69). Likewise, macrophage inhibitory factor (MIF) and intercellular adhesion molecule 1 (ICAM1) provide instructional signals to monocytes during the extravasation (70). Haitao Ge et al. have found that CD70 ablation in primary GBM cell lines can reduce the expressions of *CD44* and *SOX2* genes, inhibiting the tumor migration, growth, and ability to attract monocyte-derived M2 macrophages *in vitro* (71). In addition, kynurenine produced by GBM activates the aryl hydrocarbon receptor (AHR) in GAM cells. AHR induces the expression of CCR2 and increases the rate of recruitment of GAMs to the TME. AHR also drives the expression of KLF4 and inhibits the NF-κB-mediated inflammatory signaling in tumor associated macrophages, regulating the function of GAM cells and T-cell immunity (72).

## GAM cells’ role in glioma progression and treatment resistance

### Tumor growth and invasion

Mian-Fu Cao et al. have found that GAM-GBM cell hybrids can exist in human GBM specimens as well as in orthotopic mice models. Following the co-culture of GBM cells with BMDM, the hybrids can undergo nuclear reprogramming with a unique gene expression profile compared to their parental cells. Moreover, glioma invasion-associated genes are enriched in hybrids with higher invasiveness. More hybrids in the invasive margin of GBM have been observed in comparison to the GBM core area, suggesting that GAM -GBM cell hybrids can enhance the invasiveness of GBM cells (73). Furthermore, GAMs can improve the invasiveness of CD133^+^ tumor stem cells *via* releasing TGF-βI, thereby increasing the production of matrix metalloproteinase 9 (MMP9). The CD133^+^ glioma stem-like cells (GSLCs) isolated from xenografted gliomas in mice exhibit higher invasive potential after co-culturing with GAMs (74). Continuous autocrine stimulation of macrophages by adenosine deaminase 2 or cat eye syndrome chromosome region candidate 1 gene (CECR1) enables M2-like TAMs to stimulate MAPK and c-Jun signaling in glioma cells *via* paracrine activation, promoting tumor proliferation and migration (75). Another study has shown that GAMs can activate the ERK1/2 phosphorylation in GBM cells by secreting CCL8, thereby inducing GBM cell invasion and stem cell-like traits. Moreover, CCR1 and CCR5 are the main receptors mediating CCL8-induced biological behaviors of gliomas. Blockade of CCL8 secreted by GAMs *via* neutralizing antibodies significantly reduces glioma cell invasion (76). Furthermore, GAM-secreted abundant pleiotrophin (PTN) through its receptor PTPRZ1 stimulates glioma stem cells (GSCs) and promotes GBM malignancy through the PTN-PTPRZ1 paracrine signaling (77). The RNA regulator HuR expressed in GAMs also plays a crucial role in the tumor-promoting abilities of GAMs. Suppression of HuR-induced M1-type polarization of GAMs concomitantly reduces the expression of PDL1, increases the number of infiltrating CD4 cells (including Th1 and cytotoxic effector cells), and suppresses the tumor growth in GBM mouse models (78).

### Angiogenesis

GAMs promote tumor progression *via* regulating the angiogenesis in GBM. In GBM, CD163^+^ macrophages are widely distributed across tumor parenchyma vessels, especially between the proliferating microvessels (79). Perivascular GAMs in GBM are closely related to the density of microvessels and high expression of vascular endothelial growth factor A (VEGFA), heme oxygenase 1 (HO1), and thymidine phosphorylase (80). GAMs have been shown to enhance the vascular mimicry (VM) of glioma cells *via* upregulating the secretion of IL6 and cyclooxygenase 2 (COX2) (81, 82). Moreover, GAM-secreted IL6 promotes the angiogenesis of endothelial progenitor cells by activating the JAK-STAT signaling pathway (83). Additionally, GBM-derived C-reactive protein (CRP) induces COX2-positive GAMs to produce IL6 and IL1β, which promote endothelial cell proliferation and enhance endothelial expression of proangiogenic factors, including IL8, VEGFA, and hypoxia-inducible factor 1 alpha (HIF-1α) (84, 85). The receptor for advanced glycation end-product (RAGE) signaling in GAMs drives the GBM angiogenesis and tumor growth. Knockdown of RAGE in GBM mice models does not alter the tumor growth rate but prolongs animal survival by reducing tumor-associated inflammation (86).

### Energy metabolism

Dysregulation of energy metabolism is an emerging hallmark of tumors. There is an intricate interaction network between the metabolism of tumor cells and TME. Hypoxia, acid build-up, and immune cells in the TME can regulate the metabolism of tumor cells (87, 88). M2-type macrophages promote oxidative phosphorylation (OXPHOS) and support tumor cell proliferation by releasing large amounts of VEGFA and IL10. In response to hypoxia and lactate stimulation, macrophage-produced chemokines CCL5 and CCL18 upregulate the activities of various glycolytic factors, including lactate dehydrogenase A (LDHA) and glucose-6-phosphate dehydrogenase (G6PD), promoting glycolysis in tumor cells, which leads to the accumulation of excessive lactate in the TME and suppresses tumor immune responses (89). Jian Lu et al. have found that M2-type GAM-derived IL1β, mediated by phosphatidylinositol-3-kinase-mediated protein kinase delta (PKCδ), activates phosphorylation of the glycolytic enzyme glycerol-3-phosphate dehydrogenase (GPD2) at threonine10 (GPD2 pT10) in glioma cells. GPD2 pT10 enhances its substrate affinity and increases the catalytic rate of glycolysis in glioma cells. Inhibition of PKCδ or GPD2 pT10 in glioma cells or blocking of IL1β production by macrophages reduces glioma cells’ glycolytic rate and proliferation (90). Also, Yajuan Zhang et al. have indicated that M2-type GAMs can enhance the 3-phosphoinositide-dependent protein kinase 1 (PDPK1)-mediated phosphorylation of phosphoglyceride kinase 1 (PGK1) at threonine243 by secreting IL6. This phosphorylation promotes PGK1-catalyzed glycolysis by altering substrate affinity. Inhibition of PGK1 T243 or PDPK1 phosphorylation in tumor cells or neutralization of macrophage-derived IL6 reverses the macrophage-promoted glycolysis, proliferation, and tumorigenesis (91).

### Treatment resistance

The infiltration of GAMs and M2-type polarization in the glioma microenvironment lead to tumor immunosuppression and induces the resistance of GBM to chemoradiotherapies *via* multiple mechanisms. IL11 secreted by GAMs activates the STAT3-MYC signaling pathway, which induces the proliferation of glioma stem-like cells and confers enhanced tumorigenicity and resistance to chemotherapeutic drugs like temozolomide (TMZ) (92). Crosstalk between GBM cells and GAMs attenuates the chemotherapeutic efficacy of drugs. Studies have suggested that long non-coding (lnc) RNA TALC (lnc-TALC) is incorporated into exosomes and delivered to GAMs to promote their M2-type polarization, ultimately leading to the TMZ resistance (93). Similarly, miR-21 maturation in GAM-secreted exosomes can increase the secretion of M2-type cytokines IL6 and TGF-βI, thereby promoting the M2-type polarization of GAMs and increasing the resistance of GBM cells to the TMZ treatment (94). Furthermore, SLIT2-induced macrophage invasion and M2-type polarization in mouse GBM cells and human patient-derived GBM xenografts have exhibited enhancement of the therapeutic resistance of GBM to chemo- and immunotherapies (95). In the anti-angiogenic therapy of GBM, the recruitment and M2-type polarization of BMDMs following the administration of VEGF inhibitors constitute an immunosuppressive microenvironment, leading to treatment resistance (96). In the radiotherapy of GBM, irradiation can upregulate the expression of CSF-1R, enhancing the recruitment of BMDMs-derived GAMs, and promoting M2-type polarization, resulting in the development of radioresistance (97).

## Regulators of M1/M2-GAM activation and transformation

### Glioma cell-derived soluble molecules

Recently, multiple studies have shown that GAMs can be activated and polarized by various modulatory factors (**Table 1**), including soluble molecules derived from GBM cells, thereby promoting tumor progression and metastasis. Sonic hedgehog signaling (SHH) molecule secreted by GBM cells blocks the recruitment of CD8^+^ T-cells to the glioma microenvironment by inhibiting CXCL9 and CXCL10 to drive M2-type polarization of GAMs (98). In contrast, the CXCL16-CXCR6 axis induces the M1-type microglia while inhibiting the polarization of the M2 phenotype upon LPS or IFN-γ stimulation (99). In addition, the Na^+^/H^+^ exchanger 1 (NHE1) protein, secreted by GBM, partially promotes the M2-type activation of GAMs by stimulating the glucose metabolism and participating in the regulation of the GBM immunosuppressive microenvironment, which is recognized as a new target for improving the efficacy of immunotherapy (124, 125). Osteopontin (OPN) is a potent macrophage-derived chemokine that maintains both the M2 genotype and phenotype of GAMs. The expression level of OPN is correlated with glioma grade and GAM infiltration. Integrin αvβ5 acts as the major receptor for OPN (100). Similarly, mucin (MUC1) and polypeptide SLIT2 have also been shown to be involved in the M2-type polarization of GAMs (95, 101). Moreover, GBM cells activate CD74 expression and induce the transformation of GAMs from M1- to M2-type polarization by secreting MIFs (70).

**Table 1:** Regulators of M1/M2-GAMs activation and transformation.

M1/M2 polarization factors	Regulators	Mechanisms	References
Glioma cell-derived soluble molecules	SHH	inhibits CXCL9 and CXCL10, driving M2-polarization	(94)
	CXCL16/CXCR6 axis	inhibits LPS and IFNγ induced M2-polarization	(95)
	NHE1	Promotes M2-activation via stimulating glucose metabolism	(96, 97)
	OPN	Binds integrin αvβ5 and mediates M2-GAMS recruitment	(98)
	MUC 1	Binds siglec-9, secretes various factors ,inducing M2-like GAMs	(91)
	SLIT2	Binds ROBO1/2, activates PI3Kγ, driving M2-polarizaition	(99)
	MIF	Activates CD74, reprograming M1-GAMs to M2 phenotype	(66)
GSCs/GSLCs-derived soluble molecules	ALPs	Promotes IL12 expression ,driving M1-polarization	(100)
	ARS2/ MAGL axis	Upregulates PGE2 expression, driving M2-polarization	(101)
	WISP1	Binds α6β1, activates pAkt-Ser473, mediating recruitment and M2-polarization	(102)
	POSTN	Binds α5β3, mediating recruitment and M2-polarization	(103)
Exosomes secreted by glioma cells/GAMs	Glioma-derived H-GDES	miR-1246 expressed in H-GDES activates STAT3, inhibits NE-KB via targeting TERF2IP, driving M2-polarizatIon	(104)
	GBM-derived H-GDES	IL-6 and miR-155-3p expressed in H-GDES Inducing M2-polarization via IL-6-pSTAT3-miR-155-3p-autophage-pSTAT3 positive feedback loop	(105)
	GBex	ArgInase-1+ expressed in Gbex reprograms M1 to M2 phenotype; Inc-TALC delivered to GAMs via Incorporating into GBex, promotes complement C5 production and phophorylation of p38 MAPK, driving M2-polarozation	(89, 106)
	GAMs-derived exosomes	miR-21 expressed in GAMs upregulates IL-6, TGFB1 expression, driving M2-polarization	(90)
Immunomodulation	Tregs	Tregs inhibits CD8 T cell secreting IFNγ, maintains M2 like characteristics	(107)
	MDSCs	Hopoxic MDSCs upregulates CD45 Phosphatase and inhibits psTAT3 expression, driving tumor-suporting diferentiation of macrophages	(108)
	PD L1	PD-L1 Correlates With M2-Macrophages-Related Chemokines and is associated with signalling pathways that modulate macrophage polarization	(109)
	PD-1	PD-1 drives uncleared phagocytic material and lysosomes accumulation, promoting M2-polarizatIon	(110)
Noncoding RNAs	miR-340-5p	Targets POSTN, mediating recruiting and M2-polarization via POSTN binding receptor α5β3	(111)
	miR-106b-5p	Inhibits IRF1/IFN-β Signaling to Promote M2-Polarization	(112)
	Inc-SNHG15, mIR-627-5p	Inc-SNHG15 Inhibits tumor supressor miR-627-5p, leading to CDK66 and Sox-2 activation, driving M2-polarization	(113)
	mIR-1246, mIR-155-3p, miR-21, and Inc-TALC	See "Exosomes secreted by glioma cells/GAMs" part	(89, 90, 104, 105)
Radiotherapy and chemotherapy	SDF-1/CXCR4 axis	Iradiation induced HIF-1α upregulates SDF-1α expresion, leading to GAMs acumulation and M2-pobrization	(114, 115)
	CSF-1R	Iradiation upregulates CSF-1R expression, promoting M2-polarization	(93, 116)
	CD68, CD206	TMZ treatment upregulates M2 marker gene expression, inducing TMZ resistance	(117)
	MIF	Bevacizumab treatment inhibited MIF induced M1-polarization, driving M2-polarization	(92)
	AXL	Nivolumab treatment increased expression of AXL activation and GAMs M2-polarnation	(118)
Other factors	Autophage	Autophagy-dependent lysate-secreted KRAS protein induces M2-polarizatIon via STAT3-regulated fatty acid oxidation	(119)
	LAP	LAP inhibits string and type-1 IFN induced Ml-polarization, mediating M2-polarization	(120)
	TGF β1, integrin α5β3	TGF β1,integrin α5β3 promotes M2-polarization via Src-P13K-YAP signaling	(121)
	Hypoxia	Hypoxia upregulates POSTN expression, mediating recruitment and M2-polarization	(122)
	metabolic reprogram	Adenosine metabolism were responsible for the accumulation and M2-polarization	(123)

The GBM is highly heterogeneous at both molecular and histological levels. Not only the intratumoral heterogeneity, but GBM also exhibits a high level of intertumoral heterogeneity. Different molecular subtypes of GBM modulate different gene signatures of GAMs (102). Mesenchymal-associated glioma-associated macrophages (MA-GAMs) are the master regulators of this process, and the expression of their target genes significantly correlates with poor clinical outcomes. They are often associated with genomic aberrations in neurofibromin 1 (*NF1*) and phosphoinositide 3-kinases/mammalian target of rapamycin/Akt (PI3K-mTOR-AKT) pathway-related genes (103).

### Glioma stem cells/stem-like cells-derived soluble molecules

Cancer stem/stem-like cells are critical for cancer initiation, progression, and therapy resistance. The sc-sequencing suggests that cancer stem cells may correspond to the most malignant and proliferative glioma group of cells in gliomas, and are often the originator of other histotypes or molecularly typed glioma cells (104). Researchers have isolated a particular fraction of necrotic products spontaneously arising from glioma cells, which are morphologically and biochemically defined as autoschizis-like products (ALPs). Glioma stem cell (GSC)-derived ALPs exhibit a higher activity for the M1-GAMs polarization than those from non-GSCs (105). Furthermore, the ARS2/MAGL pathway in GSCs regulates the self-renewal and tumorigenicity of GSCs through the production of prostaglandin E2, stimulates β-catenin activation, and M2-like GAM polarization in GSCs (106), GSCs also promote the survival, and M2-like polarization of GAMs by secreting Wnt-induced signaling protein 1 (WISP1). Silencing of WISP1 has been shown to significantly disrupt the GSC maintenance and the M2-like characteristics of GAMs (107). Furthermore, GSC-secreted POSTN recruits M2-GAMs through its receptor integrin αvβ3. Knockout of POSTN in GSCs significantly reduces the GAM density, inhibits tumor growth, and increases survival in mice bearing GSCs-derived xenografts (108).

### Exosomes

Exosomes are essential elements involved in intercellular communication and TME modulation. High expression of miR-1246 in hypoxic glioma-derived exosomes (H-GDES) significantly induces the M2-type polarization of macrophages to activate the STAT3 signaling while inhibiting the NF-κB signaling pathway, which subsequently promotes glioma proliferation, migration, and invasion both *in vitro* and *in vivo* (109). Furthermore, H-GDES containing high levels of IL6 and miR-155-3p can induce M2-like macrophage polarization *via* the IL6-pSTAT3-miR-155-3p-autophagy-pSTAT3 positive feedback loop, thereby promoting the glioma progression (110). Moreover, GBM-derived arginase-1^+^ exosomes (GBex) can reprogram M1-type GAMs to M2-type and enhance the tumor-promoting functions of macrophages (126). GBM-derived lnc-TALC gets incorporated into exosomes to be delivered to GAMs, where they promote M2-type polarization and mediate TMZ resistance (93). GAM-secreted exosomes enriched in miR-21 play a role in increasing the secretion of M2-type-related cytokines IL6 and TGF-βI, promoting M2-type polarization of GAMs, and improving the resistance of GBM cells to TMZ treatment (94).

### Immune regulators

GAMs are regulated by Tregs, MDSCs, and programmed cell death 1 ligand 1 (PD-L1). IFN-γ is the major cytokine responsible for the inhibition of M2-like polarization of GAMs. Tregs inhibit IFN-γ secreted by CD8^+^T-cells, preventing the sterol regulatory element-binding protein 1 (SREBP1)-mediated activation of fatty acid synthesis in immunosuppressive M2-like GAMs. Thus, Tregs indirectly but selectively maintain the metabolic homeostasis, mitochondrial integrity, and survival of M2-like GAMs (111). MDSCs have been shown to regulate GAMs’ differentiation and promote tumor proliferation by downregulating the STAT3 level (112). PD-L1 is an unfavorable prognostic marker in GBM patients. PD-L1-mediated immunosuppression can be attributed to the infiltration and M2 polarization of GAMs (113). Furthermore, the programmed cell death 1 (PD1) protein promotes the remodeling of macrophages toward M2-type polarization, and its expression on macrophages is inversely correlated with the phagocytic potency. Blockade of the PD1-PD-L1 axis increases the rate of phagocytosis in macrophages (114).

### Noncoding RNAs

Most of the human genome is transcribed into RNA that does not code for any proteins. Any aberrant production of these non-coding RNAs (ncRNAs) is critical for the development and progression of various cancers (115). Downregulation of miR-340-5p in GBM is associated with increased tumor size, recurrence, and poor survival. MiR-340-5p directly targets POSTN, which then recruits GAMs *via* integrin αvβ3. The knockdown of miR-340-5p promotes GAM recruitment and M2 polarization *in vitro* and *in vivo* (116). In addition, miR-106b-5p has been reported to inhibit IRF1/IFN-β signaling and promote M2 polarization of GAMs (117). Likewise, lnc-SNHG15 promotes GBM tumorigenesis by inhibiting the maturation of tumor suppressor miR-627-5p, leading to activation of CDK66 and SOX-2, and M2 polarization of microglia (118). The exosome-associated ncRNAs, including miR-1246, miR-155-3p, miR-21, and lnc-TALC, also modulate the polarization of GAMs through the above-described mechanisms (93, 94, 109, 110).

### Radiotherapy and chemotherapy

Radiation has a significant impact on TME. The major changes that happen in the glioma microenvironment following the radiotherapy include decreased microvessel densities, increased ischemia-hypoxia lesions, and the accumulation of tumor-associated macrophages in these ischemia-hypoxic lesion sites. Irradiated hypoxic tissues exhibit different TME characteristics that favor the development of M2-type macrophage polarization under the regulation of tumor-secreted SDF-1α levels (127). BMDM-derived GAMs accumulate in irradiated glioma tissues after radiotherapy. These GAMs stimulate the restoration of blood flow in irradiated tumors, thereby promoting the recurrence of gliomas. SDF-1α/CXCR4 chemokine pathway drives the critical mechanism of the GAM accumulation. Hence, blocking this pathway to prevent the GAM accumulation in the TME enhances tumor response to radiation and protects irradiated tissues (119). Notably, radiotherapy upregulates CSF-1R expression and M2-type polarization to enhance the recruitment of BMDM to the TME, which promotes the development of tumor immunosuppression in gliomas (97, 120). A recent study using the combination of multi-tracer PET/MRI imaging to spatially visualize the regulation of TMZ on bone marrow-derived MDSCs and BMDMs has revealed that TMZ treatment can increase the expression of M2-type GAM marker genes in glioma microenvironment cells, which may, in turn, contribute to TMZ therapy resistance (121). Besides, VEGFR inhibitors and anti-PD-1 antibodies also lead to GAM recruitment and M2-type polarization in gliomas (96, 122).

### Other factors

Within the TME, other cellular processes, including autophagy, hypoxia, and metabolic reprogramming can also modulate the activation of GAM. Autophagy provides tumor cells with essential nutrients, nucleotides, and amino acids to promote their tumorigenic growth in the TME (123). Autophagy-dependent lysosome-secreted KRAS protein induces M2-type polarization of macrophages through the STAT3-regulated fatty acid oxidation (FAO) (128). Moreover, autophagy proteins in the bone marrow-derived glioma cells modulate LC3-associated phagocytosis (LAP) and mediate T lymphocyte-regulated immunosuppression to activate GAMs (129). M2-type polarization of GAMs correlates with angiogenic endothelial cell-macrophage and tumor cell-extracellular matrix interactions. TGF-βI and integrin αvβ3 have been shown to promote tumor-endothelial angiogenesis and M2-type polarization of GAM cells (130). Importantly, hypoxic shocks in the glioma microenvironment lead to M2-type polarization of GAMs. Both hypoxic environment and hypoxia-treated glioma cell supernatants have shown their abilities to induce M2-type polarization of GAMs. Moreover, hypoxia increases POSTN expression in glioma cells and promotes the recruitment of macrophages (131). Tumor metabolism reprogramming is crucial to the development of glioma immune tolerance. Tryptophan and adenosine metabolism have been determined to be responsible for the accumulation of Tregs and M2 macrophages, respectively, in the TME (132).

## Research progresses in targeting GAMs for the treatment of GBM

### Specific molecular inhibitors

#### CSF-1R inhibitors

The receptor tyrosine kinase (RTK) signaling pathway regulator CSF-1R is thought to play an essential role in the recruitment and differentiation of macrophages. The CSF-1R kinase inhibitors have entered clinical trials for various cancer treatments (133). Stephanie et al. used the CSF-1R inhibitor BLZ945 to target GAMs in a mouse proneural GBM model, which revealed an improved survival and regression of established tumors in treated mice. The CSF-1R inhibitors were found to slow down the intracranial growth of patient-derived glioma xenografts, also, M2-type markers were significantly reduced in surviving mouse GAMs (134). It has also been confirmed that the CSF-1R inhibitor PLX3397 interferes with the differentiation of macrophages during carcinogenesis, thus restoring the sensitivity of glioma cells to RTK inhibitors in a preclinical combination trial (135). Additionally, PLX3397 shows promising efficacy in another preclinical glioma model (136). However, to date, CSF-1R inhibitors have demonstrated limited effectiveness in GBM clinical trials. Among 37 patients with relapsed aggressive GBM, PLX3397-treated patients showed no significant improvement in progression-free survival (PFS) during the 6-month follow-up period (137). On the other hand, targeting GAMs with CSF-1R inhibitors alone may lead to antitumor responses in GBM (138). CSF-1R inhibitors may induce resistance to therapy by promoting insulin-like growth factor 1 (IGF-1) expression and activating the PI3K pathway in GAMs (139, 140). Synergistic use of CSF-1R and IGF1R inhibitors may be a more effective glioma treatment solution (140, 141).

Additionally, GBM resistance to radiotherapy may be associated with the up-regulation of CSF-1R expression, along with enhanced recruitment of BMDM-derived GAMs. Studies have shown that both CSF-1R inhibitors BLZ945 and PLX3397 can accelerate radiotherapy efficacies by blocking the radiotherapy-induced recruitment and activation of M2-type GAMs to gliomas, thereby disrupting the tumor-promoting functions of these cells in supporting glioma proliferation and regrowth (97, 120). Inhibition of CSF-1R by BLZ-945 improves the efficacy of radiation therapy in GBM. Compared to receiving radiotherapy alone, CSF-1R inhibition prevents radiotherapy-recruited monocytes from differentiating into immunosuppressive and proangiogenic GAMs. CSF-1R inhibition may be a promising strategy to overcome the hurdles of radioresistance in GBM (97, 142).

#### Chemokine receptor/ligand inhibitors

Chemokine signaling plays a crucial role in gliomagenesis, proliferation, neovascularization, metastasis, and tumor progression (143). Chemokines promote an immunosuppressive microenvironment *via* recruiting BMDMs, Tregs, and MDSCs to the TME. Immunotherapy targeting chemokines constitute one of the promising strategies for glioma treatment (144). CXCR4 is one of the critical chemokines responsible for the malignant behavior of gliomas. Lentivirus-mediated knockdown of CXCR4 showed reduced proliferation, invasion, migration, and enhanced apoptosis of glioma cells. Interestingly, a better therapeutic effect was obtained after combining CXCR4 knockdown with miR-21 expression (145). A novel inhibitor of CXCR4, named peptide R, reduced the metabolic activity, proliferation, and migration capacity of U87MG cells *in vitro* and inhibited tumor growth in an orthotopic GBM mouse model (146). Another brain-penetrating CXCR4 antagonist, PRX177561, enhanced the efficacy of anti-angiogenic therapy in GBM after co-administration with bevacizumab or sunitinib and was considered an effective complementary strategy to anti-angiogenic treatments (147). Studies have shown that combined use of the CXCR2 inhibitor SB225002 and TMZ can reduce the TMZ-induced activation of the IL8-CXCL2-CXCR2 signaling axis, inhibit tumor angiogenesis and GAM infiltration, and enhance TMZ chemotherapy efficacy (148, 149). Furthermore, monoclonal antibodies targeting mouse and/or human CCL2 show prolonged survival in C57BL/6 mice bearing intracranial GL261 gliomas, which coincides with the reduction of GAMs and MDSCs in the TME and may enhance the therapeutic benefits of TMZ (150). In addition, the combination of chemokine- and PD1-targeted immunotherapy has shown a certain therapeutic potential. Compared with the anti-PD1 monotherapy, anti-CCR2 or anti-CXCR4 therapy combination can achieve better therapeutic efficacy in mouse glioma models (151, 152).

#### VEGF inhibitors

Anti-angiogenic therapy is one of the promising options for the treatment of gliomas. Unfortunately, despite the initial efficacy of anti-angiogenic therapies, the effective durations of these drugs are limited, and drug resistance following long-term use is almost inevitable (138). In preclinical studies, the VEGF inhibitor bevacizumab, as a single agent for GBM, only showed benefits in imaging and clinical responses but had no significant effect on the PFS (153). An essential mechanism of GBM resistance to VEGF inhibitors lies in the recruitment and M2-type polarization of BMDM-derived GAMs after the drug administration. Compared with BRM, BMDM-derived GAMs preferentially lead to treatment resistance. VEGF-targeting glioma immunotherapy needs to overcome the immunosuppressive microenvironment supported by the BMDM-derived GAMs (96). In terms of mechanism, the anti-VEGF/VEGFR therapy upregulates the expression of CXCR4, SDF-1α, and TGF-βI, leading to the recruitment of BMDMs and M2-polarization. Therefore, multi-drug regimens may be more appropriate during the anti-vascular production treatment (147). The good news is that a novel SDF-1α inhibitor, olaptesed pegol (OLA-PEG), has been shown to reduce the recruitment and activation of GAMs by anti-VEGF therapy and enhance its antitumor efficacy in GBM (154). Moreover, the dual inhibitory effect of VEGFR/ANG2 on the M1-type polarization of GAMs increases the ratio of M1 to M2 macrophages, thereby extending the survival of preclinical GBM mice (155). These findings suggest a new therapeutic strategy to suppress the recruitment of VEGFR-induced GAMs, and M2-type polarization by integrating anti-ANG2, anti-SDF-1α, and anti-CXCR4 therapies, which is expected to overcome the limitations of anti-VEGFR monotherapy in GBM patients.

#### Anti-PD1/PD-L1 immunotherapy

Immunotherapy targeting the PD1/PD-L1 axis offers novel therapeutic options for the treatment of many cancers. However, durable antitumor responses have only been observed in a few patients, and preclinical as well as clinical studies have demonstrated that PD1/PD-L1 blockade can result in abnormalities in the TME that reduces the efficacy of anti-PD1/PD-L1 therapy (156, 157). Studies have shown that anti-PD1 monoclonal antibody treatment induces GAM polarization to the M1 phenotype, significantly inhibiting the intracranial tumor growth in GBM mice. The therapeutic effect of anti-PD1 may be regulated by the innate immune system, independent of CD8^+^T-cell-mediated pathways (158). What is inspiring is that multiple studies have adopted multi-drug combinations to improve the treatment efficacy of anti-PD1 therapy. The combined use of the p38MAPK inhibitor and PD-L1 antibody effectively prolonged the survival rate of TMZ-resistant GBM hosts and significantly reduced BMDM accumulation and PD-L1 abundance in BRM (159). Another study showed that treatment of GSC-derived mouse GBM tumors with nivolumab, an anti-PD1 antibody, resulted in the recruitment of intratumoral GAMs and activation of AXL, an RTK. Combining the AXL inhibitor BGB324 with nivolumab also prolonged the survival in GBM tumor-bearing mice (122). Similarly, co-administration of the CSF-1R inhibitor BLZ945 blocked the M2-type polarization of CD163^+^ GAMs and enhanced the function of CD154^+^ CD8^+^ T-cells and apoptosis of glioma cells, thereby enhancing the efficacy of nivolumab (160). In addition, the combined use of IL6 inhibitor and CD40 agonist reversed M2-type GAM-mediated tumor immunosuppression. These small molecule inhibitors sensitized tumors to the immune checkpoint inhibitor combination of anti-PD1 plus anti-CTLA4 antibodies and prolonged the survival of animals in two syngeneic GBM models (161). With the development of gene-editing technologies, CRISPR/Cas9-mediated PD-L1 knockout using dual single-guide RNAs (sgRNAs) and homology-directed repair (HDR) template is also a promising therapeutic option (162).

### Other chemical drugs

Nina Xue et al. have reported that chlorogenic acid (CGA) treatment increases the LPS/IFN-γ-induced expression of M1-type GAM markers like iNOS, MHCII, and CD11c, decreases IL4-induced expression of M2 markers Arg and CD206, suggesting that CGA may be a potential therapeutic option to inhibit glioma growth by promoting M1-type and inhibiting M2-type polarizations of GAMs (163). Sanford PC Hsu et al. could reduce the M2-type polarization of GAMs and increase phagocytic capacity and lipid droplet accumulation by combined RQ therapy with rapamycin (R) and hydroxychloroquine (Q). Whereas the RQ treatment reduced the expression levels of CD47 and SIRPα on tumor cells and macrophages in co-culture experiments. After the RQ treatment, the intratumoral ratios of M1 to M2 and CD8^+^ to CD4^+^ were significantly increased in the intracranial GL261 tumor models. Moreover, the combination of RQ and anti-PD1 therapies demonstrated synergistic efficacies (164). Jie Li et al. have shown that inhibitors targeting PI3Kγ reduce the GAM-associated IL11 secretion in the GBM microenvironment *via* pharmacological inhibition and enhance TMZ therapy efficacy in orthotopic GBM mice (92).

### Applications of liposomes and nanomaterials

The existence of the BBB restricts the penetration of drugs from the bloodstream to the CNS. Therefore, it constitutes the main obstacle that therapeutics must overcome to enter brain tumors. Ensuring that the drug is fully penetrated the brain is essentially the decisive factor in the drug efficacy determination (165). Moreover, the application of ultrasound to increase the permeability of the BBB could improve immunotherapy efficacy (166). Multiple studies have applied liposomes and nanomaterials as drug carriers through advanced materials technology to achieve biomimetic delivery of therapeutic drugs across the BBB. Pengfei Zhao et al. have designed a bipolar-modified albumin nanoparticle, which achieved the bionic delivery of drugs to the gum tumor area through BBB. The therapeutic must reach its targeted lesion sites (M2-type GAMs) immediately following its penetration through the BBB. This drug delivery system could successfully reprogram GAMs from the M2- to M1-type polarization, thereby effectively inhibiting glioma cell proliferation (167). Feng Zhang et al. have developed a glioma-targeted infusion-based nanocarrier containing interferon regulatory factor 5 (IRF5)-encoding mRNA along with its activating kinase IKKβ that shows a reversal of the immunosuppressive microenvironment of gliomas. These drug-loaded nanoparticles can reprogram GAMs to an M1 phenotype that induces antitumor immunity and promotes tumor regression (168). The study by Xiaopeng Mo et al. has reported the co-encapsulation of simvastatin and fenretinide into D-α-tocopheryl polyethylene glycol succinate (TPGS)-TAT (a cell-penetrating peptide)-embedded lactoferrin nanoparticle system for brain-targeted biomimetic delivery *via* the LRP-1 receptor. The results suggest that lactoferrin nanoparticles can repolarize GAMs from the M2 phenotype to M1 by modulating the STAT6 pathway and induce reactive oxygen species (ROS)-mediated mitochondrial apoptosis by inhibiting the Ras/Raf/p-ERK pathway in glioma cells (169).

Nasha Qiu et al. have designed an IL12 delivery vector, which can embed IL12 expression plasmid to form lipid complexes to effectively transfect tumor cells and macrophages and make them IL12 production factories. This strategy has been shown to improve the M1/M2 macrophage ratio, thereby activating antitumor immune responses and remodeling the TME (170). Zening Zheng et al. have developed a brain-targeted liposome and disulfiram/copper cassette system (CDX-LIPO). CDX-LIPO activates tumor-infiltrating macrophages, dendritic cells, prime T-cells, and natural killer (NK) cells. Moreover, it can trigger tumor cell autophagy, inducing immunogenic cell death. CDX-LIPO also promotes M1-type polarization of GAMs and mTOR-mediated reprogramming of glucose metabolism in gliomas, leading to antitumor immunity and tumor regression (171).

## Conclusion

**Figure 2:**
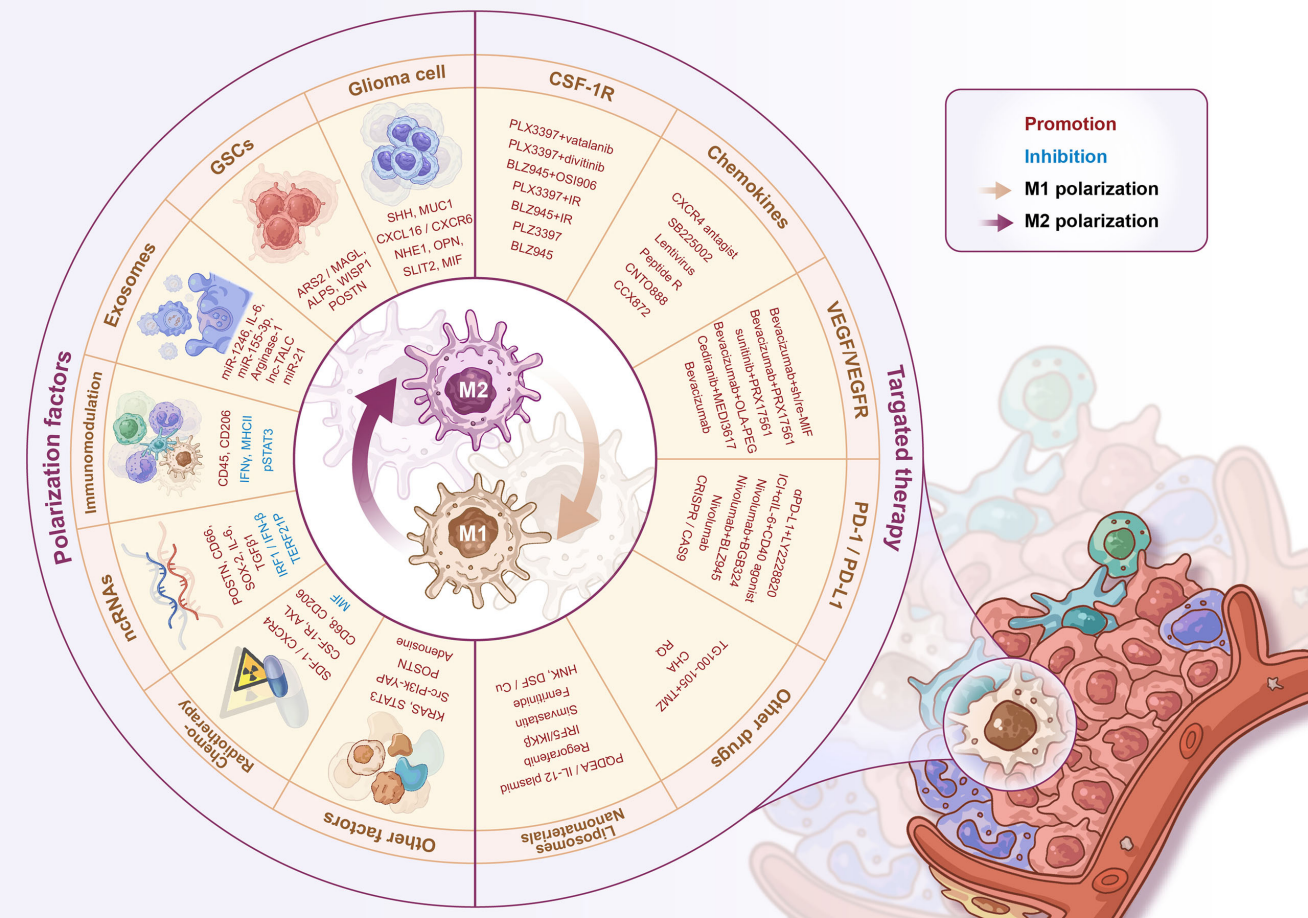
Regulators of M1-M2 polarization and GAMs’ targeted therapy. The regulators of GAMs polarization include a variety of soluble factors secreted by glioma cells/stem cells, exosomes, immune-related molecules, non-coding RNAs, radio-chemotherapy and some other proteins, signaling pathways, etc. The GAMs targeted therapy strategies include CSF-1R inhibitors, chemokine inhibitors, anti-angiogenic therapy, PD-1/PD-L1 inhibitors, and novel liposome/nanomaterial delivery systems for drug delivery.

The immunotherapy of tumor-associated macrophages has recently attracted the attention of clinicians and researchers. As a highly malignant solid tumor, gliomas have their unique pathological characteristics involving distinct TME and tumor-associated macrophage populations. Gliomas are considered immunologically “cold tumors”. There is a high degree of heterogeneity between different subtypes of glioma tumors and within the tumor itself, as well as the unique spatial structure BBBs under pathological conditions. These factors pose enormous challenges to the treatment of gliomas. As a new option for glioma treatment, immunotherapy combined with standard therapy could be a promising solution to improve the survival of glioma patients (172). In-depth exploration of the glioma tumor immune microenvironment and the macrophage recruitment and activation mechanisms are the basis for developing novel glioma immunotherapy strategies. Complex crosstalks and regulatory networks between GAMs and tumor cells contribute to the severe malignancy of gliomas and subsequent treatment resistance. Drug development targeting the critical molecules in the process of GAM recruitment and activation can reduce the accumulation of BMDM-derived GAMs, and reprogram the polarization pattern of GAMs to increase the M1/M2 macrophage ratio, ultimately reversing the tumor immunosuppressive microenvironment in gliomas (**Figure 2**).

Presently, various immunotherapy drugs targeting GAMs, such as CSF-1R inhibitors, PD-L1 antibodies, VEGF inhibitors, and some chemokine inhibitors have achieved a certain degree of success in the experimental stages using *in vitro* and *in vivo* xenograft models. It is worth noting that the high heterogeneity of glioma indicates that the efficacy of single-drug therapy is often limited and is prone to the development of drug resistance. The multi-drug combination therapy scheme is expected to overcome the side effects and treatment resistance brought by single-drug treatment. Preclinical trials using different compositions of a therapeutic regimen such as CSF-1R inhibitors combined with IGF1 inhibitors (140), VEGFR inhibitors combined with ANG2 inhibitors (155), CXCR2 inhibitors combined with TMZ (148, 149), and PD-L1 antibody combined with p38MAPK inhibitors, AXL inhibitors or CSF-1R inhibitors (122, 159, 160) have achieved better therapeutic effects than their monotherapies. Notably, the application of materials technology such as liposomes and nanomaterials is expected to solve the problem of drug penetration across the BBB and CNS, ensuring a selective targeting and high bioavailability of therapeutic anticancer drugs in the TME, thus providing a new option for the glioma treatment **(** **Table 2** **)**.

**Table 2:** Research progress of targeting GAMs in the treatment of GBM.

GAMs targets	Inhibitors	Medication regimen	Research progress	References
CSF-1R	BLZ945	Monotherapy	Promote mouse xenograft model survival, regress established tumor, and decreased M2 markers expression	(129)
	PLX 3397	Monotherapy	PLX3397 decreased GAMs accumulation and invasiveness GBM cells in mouse models, slowed tumor growth in mouse models. PLX3397 was well tolerated, readily crossed the BBB but showed no efficacy in GBM patients	(131, 132)
	PLX3397	PLX3397 + vatalanib/dovitinib (tyrosine kinase inhibitors)	PLX2397 inhibits CSF1R phosphorylation, abrogates GAMs M2-polarization, promoting vatalanib and dovitinib therapy efficacy	(130)
	BLZ945	BLZ945+0S1906 (IGF-1R inhibitor)	BLZ945 monotherapy promotes drug resistance via IGF-1R induced PI3K activation, Synergistic use of BLZ945 and 0S1906 prolonged mouse models survival	(134, 135)
	PLX 3397	PLX3397+ IR	IR increased CSF-1R ligand expression and increased CD11b BMDMs in the tumors. PLX3397 both depleted CD11b+ cells and sensitized intracranial tumors to IR	(93)
	BLZ945	BLZ945+IR	Combined treatment is more effective than either therapy alone in GBM mouse models, and delays glioma relapse	(116, 137)
Chemokines	Lentivirus	sh-CXCR4 + pLenti-anti-miR-21	Double-Targeted Knockdown of miR-21 and CXCR4 Inhibited Malignant Glioma Progression via downregulating PI3K/AKT and Raf/MEK/ERK Pathways	(140)
	Peptide R	Monotherapy	Peptide R targeting CXCR4 reduced tumor cellularity in vitro, promoted M1 features of GAMs and astrogliosis in orthotopic mouse models	(141)
	SB225002	SB225002 (CXCR2 inhibitor) + TMZ	SB225002 inhibits tumor angiogenesis and Infiltration of GAMs, enhanced anti-tumoral effects were observed after combi-therapy with TMZ	(143, 144)
	CNT0888	CNT0888 (CCL2 monoclonal antibodies) +TMZ	CNT0888 promotes mouse models survival via decreasing GAMs and MDSCs, CNT0888 enhanced TMZ efficacy after co-administration	(145)
	CCX872	CCX872 (CCR2 antagoist) + PD-1 Inhibitor	CCX872 promotes mouse models survival via decreasing MDSCs, CCX872 enhanced anti-PD-1 efficacy after co-administration	(146)
	CXCR4 antagoist	CXCR4 antagoist + PD-1 inhibitor	Co-administration showed significant survival benefit than anti-PD-1 monotherapy, decreased MDSCs and GAMs accumulation in the mouse models	(147)
VEGF/VEGFR	Bevacizumab	Monotherapy	Bevacizumab improved radiographic response and clinical benefit to GBM patients, but showed no advantage in PSF compared with historical controls	(148)
	Bevacizumab	Bevacizumab + sh /recombinant MIF	Bevacizumab monotherapy reduces MIF expression, drives GAMs recruitment and M2-polariztion, mediating therapy resistance	(92)
	Bevacizumab, sunitinib	Bevacizumab/sunitinib + PRX17561(CXCR4 inhibitor)	Co-administration of PRX177561 with bevacizumab/sunitinib inhibited tumor growth and reduced the inflammation, increased efficacy of bevacizumab/sunitinib	(142)
	Bevacizumab	Bevacizumab + OLA-PEG (SDF-I inhibitor)	Combination therapy significantly promotes GBM mouse models survival compare with bevacizumab monotherapy, OLA-PEG decreased M2-GAMs accumulation	(149)
	Cediranib	Cediranib + MEDI3617 (Ang-2 antibody)	Dual anti-VEGFR/Ang-2 therapy extends survival, improves vessel normalization, alters GAMs polarization to M1-type compared with cediranib monotherapy	(150)
PD-1/PDL-1	Nivolumab	Monotherapy	Neoadjuvant nivolumab resulted in enhanced expression of chemokine transcripts, higher immune cell infiltration, but no significant clinical benefit. Nivolumab induce apoptosis of microglia through antibody-dependent cellular cytotoxicity	(152, 153)
	PD-L1 antibody	PD-L1 antibody +LY2228820 (p38 MAPK inhibitor)	Combination therapy effectively prolongs the survivals of TMZ-resistant GBM-bearing hosts, reduces the accumulation of BMDMs and PD-L1 abundances of BRM	(154)
	Nivolumab	Nivolumab +BGB324 (AXL inhibitor)	Nivolumab treatment increased intratumoral GAMs accumulation and AXL activation. Combinatorial therapy effectively prolonged the survival of GBM mouse models	(118)
	Nivolumab	Nivolumab + BLZ945 (CSF-1R inhibitor)	BLZ945 ablates CD163+ M2-GAMs and strengthened CD154+CD8+ Tell functionality and GBM apoptosis, enhancing Nivolumab efficacy	(155)
	ICIs	ICIs + IL-6 antibody & CD40 agonist	IL-6 neutralization and CD40 stimulation synergistically reduces GAMs-mediated immune suppression and enhances T-cell infiltration and activation in GBM, sensitizes GBM to immune checkpoint blockade	(156)
	CRISPR/Cas9	dual-sgRNAs + HDR template	PD-L1 deletion by gene-editing system increases TNF- α and decreases IL-4 secretions, repolarizes GAMs to M1 phenotype, inhibiting GBM progression.	(157)
Other drugs	CHA	Monotherapy	CHA treatment increased MI markers (INOS, MHC II, CD11c) expression and reduced M2 markers (Arg, 032061 expression, inhibiting tumor cells growth	(158)
	RQ	Rapamycin (R)+ hydroxychloroquine (Q)	RQ treatment decreased M2-polarization, increased the phagocytic ability and lipid droplets accumulation. Enhanced the intra-tumoral M1 /M2 ratio	(159)
	TG100-105	TG100-105 + TMZ	TG100-105 inhibited IL-11 induced STAT3-MYC signaling pathway activation, decreased GAMs accumulation, and enhanced TMZ efficacy	(88)
Liposomes/ Nanomaterials	Regorafenib	Disulfiram/copper complex	The albumin nanoparticles modified with dual ligands efficiently passed through the BBB and achieving biomimetic delivery to glioma and GAMs, inhibited the glioma cell proliferation and reprogrammed M2-GAMs to M1 phenotype.	(162)
	IRF5, IKK *β*	Nanoparticles	The nanoparticles formulated with mRNAs encoding IL-5 and IKK *β* reverse the immunosuppressive, tumor-supporting state of GAMs and reprogram them to M1 phenotype that induces anti-tumor immunity and promotes tumor regression	(163)
	Simvastatin, fenretinide	TPGS-TAT-embedded lactoferrin nanoparticle	Lactoferrin nanoparticles repolarize GAM from M2 phenotype to M1 by modulating STAT6 pathway and induce ROS-mediated mitochondrial/ apoptosis by inhibiting Ras/Raf/p-Erk pathway in glioma cells	(164)
	PQDEA, IL-12 plasmid	APEG-LPs/pIL12	APEG-LPs/pIL12 transfected GAMs and tumor cells to produce IL12 and converted M2 GAMs to M1 type, recruited T cells and NK cells, and reduced the Tregs population, leading to antitumor effect and prolonging mouse models survival	(165)
	HNK, DSF / Cu	CDX-LIPO	CDX-LIPO activates tumor-infiltrating macrophages and dendritic cells, promotes M1-polarization leading to antitumor immunity and tumor regression. CDX-LIPO promotes mTOR-mediated reprogramming of glucose metabolism in gliomas	(166)

Regrettably, in these studies, liposomes and nanomaterials have been used more frequently as carriers to achieve biomimetic delivery of therapeutic drugs through the BBB. Still, the medicinal drugs loaded onto the carriers are not widely used in the targeted inhibition of key molecular switches in GAMs (such as CSF-1R, VEGF, PD-1, etc.). Further comprehensive investigations are urgently needed to achieve therapeutic breakthroughs in drug research and development through better cooperation between material scientists, immunologists, and clinicians in the future to develop effective immunotherapy or adjuvant therapy for glioma patients. Finally, with the development of single-cell sequencing technology in recent years, researchers have been able to better analyze and understand the spatial heterogeneity of glioma immune cells, where simplified M1 and M2 profiling may not be enough to fully represent the glioma microenvironment in the patients. Due to the complex immune status of macrophages, the emergence of new immune typing in the future may lead to more detailed and effective treatment strategies.

## Author contributions

CX and MX wrote the article. XL, LX, JS, and QZ participated in literature collection and organization. CW, QZ, and XY participated in figures and tables generation. CX and YT provided critical perspectives on the article ideas. CF and YT fund the study and provide article revision. All authors contributed to the article and approved the submitted version.

## Fundings

This study was supported by National Natural Science Foundation of China (NSFC, No.82172660), The Hebei Provincial Government subsidized the clinical medicine outstanding talent training project (No.361007), Hebei Province Graduate Innovation Project (No.HBU2022bs012).

## Conflict of interest

The authors declare that the research was conducted in the absence of any commercial or financial relationships that could be construed as a potential conflict of interest.

## Publisher’s note

All claims expressed in this article are solely those of the authors and do not necessarily represent those of their affiliated organizations, or those of the publisher, the editors and the reviewers. Any product that may be evaluated in this article, or claim that may be made by its manufacturer, is not guaranteed or endorsed by the publisher.
